# Involuntary and patient-initiated delays in medical care during the COVID-19 pandemic

**DOI:** 10.1093/haschl/qxad057

**Published:** 2023-11-02

**Authors:** Erin T Bronchetti, Ellen B Magenheim, Ethan K Bergmann

**Affiliations:** Department of Economics, Swarthmore College, Swarthmore, PA 19081, United States; Department of Economics, Swarthmore College, Swarthmore, PA 19081, United States; Industrial Relations Section, Princeton University, Princeton, NJ 08544, United States

**Keywords:** COVID-19, delayed and forgone medical care, preventive care

## Abstract

This paper uses data from a new, nationally representative survey to study delays in non–COVID-related medical care among US adults during the COVID-19 pandemic. We expand on prior research by taking a comprehensive look at the many reasons patients may have experienced delays in medical care and by studying the longer-run implications of these delays for patients’ self-reported health, use of telemedicine, feelings of regret, and likelihood of delaying care again in the future. Classifying delays in care broadly as involuntary (those due to availability or “supply-side” constraints) or patient-initiated (those due to patient concerns or “demand-side” constraints), we document important differences across demographic groups in the propensity to delay care for these reasons. In contrast to most prior work on this topic, our analyses can disentangle differences in the likelihood of delaying care from differences in pre-pandemic care-seeking behavior. We also demonstrate that the types of medical care that were delayed during the pandemic differed based on whether the delay was involuntary or patient-initiated, as did the duration of the delays and their associations with self-reported health, telemedicine use, and feelings of regret.

## Introduction

The early months of the COVID-19 pandemic in the United States were marked by substantial reductions in the consumption of non–COVID-related medical care, with many Americans reporting that they had delayed or gone without routine and ambulatory medical care, as well as urgent and emergency care.^1-4^ Hospital data corroborate these self-reports of delayed or forgone care, showing significant decreases in hospital admissions, overall emergency department (ED) visits,^5^ and even ED visits for acute medical concerns like heart attack, stroke, and hyperglycemic crisis.^6^ Doctors and public health policymakers have expressed concern that these delays in preventive and ambulatory care are likely to have negative and long-lasting consequences. While it is too early to fully assess the morbidity and mortality impacts of some delayed and forgone care, recent work suggests that forgone cancer screenings led to many missed diagnoses,^7^ and early survey evidence finds that many patients felt their health deteriorated as a result of delaying.^4,8^ More than 3 years from the start of the pandemic, however, we still lack a comprehensive understanding of why these delays occurred, which types of patients were most affected, and how patients responded to these disruptions in care over time.

Existing research explaining pandemic-related delays in care has considered both supply and demand factors as playing important roles. In the spring of 2020, many doctors’ offices and health care facilities in the United States closed or postponed appointments, either due to capacity constraints or to local stay-at-home orders.^3^ Such disruptions are described by Callison and Ward^9^ as “involuntary” delays in care, because they left patients who were seeking care unable to access it. Their study examines patient characteristics that were associated with a greater likelihood of such delays, such as fair or poor health, having a college education, or having health insurance coverage. But, as the authors note, without data on individuals’ baseline consumption of health care, they are unable to determine whether these associations simply reflect that these groups of patients had a higher propensity to consume medical care prior to the pandemic.

The evidence is more limited when it comes to delays in care that originated on the patient side. Patients may have made active decisions to delay care in an effort to avoid exposure to COVID-19, and/or they may have faced logistical, financial, or other bandwidth constraints that prevented them from obtaining care at the time it was needed. For example, without their usual child care arrangements, parents may have been unable to attend their own appointments. In addition, the financial consequences of the pandemic (eg, the loss of employment, health insurance, and/or the inflexibility of insurance plans to altered methods of care) affected the affordability of health care for some households. The extant literature provides some insight into the roles of concerns about COVID-19 transmission^8,10^ and financial constraints in explaining delays in care.^10^ However, these studies focus on the earliest few months of the pandemic, lack information about baseline health care consumption, and do not separately examine the roles of different patient characteristics (eg, income, health insurance status, educational attainment) in describing which groups were most likely to experience delays in care.

Our study aims to provide a more complete picture of the causes and some of the consequences associated with delays in needed medical care during the COVID-19 pandemic. To do so, we used data from a new, nationally representative survey (*n* = 1480) that we designed and fielded specifically for this purpose. Our contributions to the literature are 3-fold. First, existing studies of delayed or forgone care have focused on just 1 or 2 explanations for why patients delayed care, and many do not distinguish between involuntary and patient-initiated delays in care.^1,8^ In contrast, our study sheds light on the relative importance of many different factors, including limited availability of care, concerns about contracting COVID-19, and financial difficulties, logistical constraints, and feeling overwhelmed with other obligations and responsibilities. Second, we provide new evidence on the types of care affected by involuntary and patient-initiated delays, the duration of their avoidance of in-person care, their use of telemedicine during the pandemic, and the impacts of care delays on their health. Finally, a key limitation in most existing studies is a lack of information on individuals’ baseline (pre-pandemic) consumption of medical care. Without such information, it is impossible to disentangle the higher prevalence of care delay among some groups of respondents vs others from differences in the groups’ baseline propensities to seek medical care. For example, a finding that lower-income people were less likely to experience pandemic-related delays in care may simply reflect that those with lower incomes were hardly consuming any medical care to begin with. To address this, our survey data include detailed information on respondents’ pre-pandemic care-seeking rates for both preventive and ambulatory care, as well as their access to a usual place of care.

## Data and methods

### Survey data

In the fall of 2021, we contracted with Qualtrics to field our survey among a diverse pool of respondents, aiming for a nationally representative sample. The survey was conducted from October 15, 2021, to November 15, 2021, using a quota system to achieve representativeness on gender, age, race, household income, education, and geographic region. The analysis sample contains 1480 respondents who completed the survey in full. Table 1 reports means for our full sample and for the subgroups of respondents who did and did not delay medical care during the pandemic and compares these with national estimates from the American Community Survey and the National Health Interview Survey. The only striking difference between our sample means and the national estimates is that respondents in our sample are disproportionately likely to have attended some college without earning a 4-year degree. The Qualtrics quota on educational attainment for our survey enforced national representation on “some college or more” but did not distinguish between those who earned 4-year degrees and those who did not.

**Table 1.** Mean characteristics of survey respondents.

	(1) Full sample	(2) No delay in care	(3) Delay in care	(4) *P* value (col 2 = col 3)	(5) National estimate
Demographics					
Age 18–24 y	0.128	0.110	0.162	.004	0.117
Age 25–34 y	0.164	0.149	0.192	.035	0.174
Age 35–44 y	0.199	0.188	0.219	.150	0.170
Age 45–54 y	0.130	0.140	0.111	.116	0.157
Age 55–64 y	0.157	0.150	0.170	.314	0.166
Age ≥65 y	0.224	0.264	0.146	<.001	0.216
Woman	0.534	0.521	0.561	.136	0.510
White	0.666	0.674	0.652	.409	0.636
Black	0.157	0.154	0.162	.686	0.118
Asian or Pacific Islander	0.061	0.065	0.055	.478	0.060
Other race	0.063	0.054	0.079	.064	0.078
Hispanic or Latino ethnicity	0.180	0.171	0.198	.214	0.169
Education					
High school degree or less	0.271	0.280	0.253	.262	0.380
Some college (<4 y)	0.506	0.521	0.478	.123	0.295
Bachelor's degree or higher	0.223	0.199	0.269	.002	0.325
Household characteristics					
Income <$50 000	0.347	0.345	0.350	.853	0.373
Income $50 000–$150 000	0.543	0.548	0.534	.591	0.456
Income >$150 000	0.110	0.107	0.117	.567	0.172
No health insurance^a^	0.072	0.080	0.057	.109	0.103
Married or cohabiting	0.549	0.557	0.534	.381	0.775
Number of people in household^a^	2.786	2.627	3.093	<.001	2.476
Household includes children <6 y	0.159	0.113	0.249	<.001	0.123
Household includes children 6–17 y	0.169	0.168	0.170	.939	0.172
Household includes adults >65 y	0.246	0.235	0.267	.179	0.313
Health status					
No. of pre-existing conditions^a^	1.53	1.41	1.74	<.001	—
Fair or poor health^b^	0.186	0.171	0.213	.049	0.136
Baseline health care utilization					
At least 1 well visit per year^c^	0.786	0.763	0.832	.002	0.816
Sick visits never or less than once per year^c^	0.467	0.545	0.316	<.001	—
Sick visits 1–2 times per year^c^	0.398	0.363	0.464	<.001	—
Sick visits 3+ times per year^c^	0.135	0.091	0.219	<.001	—
No usual place for care^b^	0.239	0.258	0.202	.016	0.099
Observations	1480	974	506	—	—

Estimates reflect means for full sample of survey respondents (column 1), means for respondents who did not experience a pandemic-related delay in medical care (column 2), and means for respondents who experienced a pandemic-related delay in medical care for any reason (column 3). Most estimates in column 5 come from the 2021 American Community Survey. Estimates for health status and health care utilization come from the 2019 National Health Interview Survey.

^a^The reference period for these variables is March through December 2020.

^b^The reference period for these variables is January 2020, prior to the pandemic.

^c^The reference period for these variables is “a typical year prior to the pandemic.”

More than one-third (34%, *n* = 506) of our sample reports having delayed or gone without medical care since March 2020 for reasons related to the pandemic. Specifically, these respondents answered in the affirmative the question, “Since it began in March 2020, did the COVID-19 pandemic ever lead you to delay or forgo getting medical care (for medical conditions NOT related to treating COVID-19)?” On average, those who experienced delays in care were younger, lived in larger households, were more likely to live with young children, were more likely to have a college degree, and had more pre-existing health conditions than those who did not. (We note that the fraction of our overall sample that reports having at least 1 chronic condition is quite high, exceeding 90%. On average, survey respondents reported having 1.54 chronic conditions that were associated with a high risk of illness from COVID-19 [see Appendix Table A1 for the list of conditions] or “[an]other serious chronic condition.”)

Of those who delayed or went without needed medical care, 42% reported that limited availability of care was the only reason for their delay. The remaining 58% of those who reported having delayed or gone without care experienced what we refer to as “patient-initiated” delays in care. (Appendix Table A2 reports means for our sample by the type of delay experienced.) To paint a more comprehensive picture of why these patients chose to delay care, we surveyed these respondents about the importance of several factors, including concerns about COVID-19 exposure, financial constraints, logistical constraints, and feeling overwhelmed by other life obligations.

### Methods

We studied the determinants of involuntary and patient-initiated delays in care using a descriptive regression that estimates relationships between delay in medical care and individuals’ characteristics, including their age, gender, education, race, Hispanic ethnicity, marital status, the presence of children or elderly members in the household, household income, political leanings, and COVID-19 conditions in their area. We measured individuals’ political leanings by asking them how they typically vote in elections, with possible responses of “Mostly vote Republican,” “Mostly vote Democrat,” “Independent or equally Republican or Democrat,” “Don’t usually vote,” or “Prefer not to answer.” As a control for COVID-19 conditions in an individual's county, we included the cumulative number of COVID-19 cases over the time period prior to vaccines, from March 1 through December 30, 2020, divided by the county population.

Our main estimates are from linear probability models in which the dependent variable is an indicator for having experienced a delay in medical care. In the case of involuntary delays, the indicator equals 1 if the individual reports that the pandemic caused them to delay or forgo non–COVID-related medical care during the period from March 2020 to the time of the survey and indicates that availability of care was the only reason for the delay. With regard to patient-initiated delays, the relevant indicators reflect individuals having delayed or gone without care for reasons other than availability of care and indicating a given factor (eg, concerns about COVID-19 exposure, financial difficulties, logistical constraints, or feelings of being overwhelmed by life obligations) was an important reason for their delay. That is, these outcome variables equal zero if the respondent did not initiate a delay in care or initiated a delay but did not list the relevant factor as a reason for doing so.

Unlike most prior research, we estimated these determinants of pandemic-related delays in medical care while controlling for pre-pandemic access to, and utilization of, health care. We controlled for differences in individuals’ baseline propensity to consume health care by including pre-pandemic measures of well-visit and sick-visit frequency, along with an indicator for whether the individual lacked a usual place for ambulatory care (before the pandemic). In analyses not displayed here, we probed the robustness of these results by dropping the control for local COVID-19 conditions, including state fixed effects, and limiting the sample by eliminating groups of respondents who spent relatively little time on the survey or those who failed attention checks late in the survey, after key questions had been asked. In all cases, the results were qualitatively similar to those described below (see Appendix Tables A3–A6).

## Results

### Determinants of involuntary delays in care

We first revisited the question of which patients were most impacted by involuntary delays in medical care during the pandemic, extending on the approach in Callison and Ward^9^ by including additional controls for household income and household composition, respondents’ political leanings, county-level per-capita COVID-19 cases, and several variables that reflect a respondent's pre-pandemic propensity to consume medical care.

The results in column 1 of Table 2 indicate that those with higher educational attainment were more likely to experience availability-related delays in medical care, as were those from higher-income households. Given that our regressions controlled for baseline health care consumption, these findings cannot be explained by such households consuming more care, generally, and thus being mechanically more likely to experience delays. It could be that more highly educated and higher-income individuals were more likely to live in areas in which providers faced significant capacity constraints or COVID-19 shutdowns lasted longer. Or it may be that these individuals are more likely to consume certain types of care (eg, musculoskeletal surgeries, diagnostic imaging, or dermatological care^11-13^) that were more likely to be delayed or canceled during the pandemic.^14^

**Table 2.** Determinants of pandemic-related delays in medical care.

	Involuntary delays	Patient-initiated delays		
	(1) Availability was only reason	(2) Any patient-initiated delay	(3) Concerns re: contracting COVID-19	(4) Financial, logistical, overwhelmed
Age 25–44 y	−0.037 (0.032)	−0.058 (0.037)	−0.064* (0.036)	−0.055 (0.035)
Age 45–64 y	0.021 (0.034)	−0.075* (0.039)	−0.084** (0.038)	−0.110*** (0.037)
Age ≥65 y	−0.046 (0.037)	−0.109** (0.043)	−0.111*** (0.042)	−0.142*** (0.040)
Woman	−0.016 (0.019)	0.044** (0.022)	−0.044** (0.021)	0.021 (0.020)
Some college (<4 y)	0.029 (0.022)	−0.016 (0.025)	−0.003 (0.024)	0.002 (0.023)
Bachelor's degree or more	0.055** (0.026)	0.015 (0.031)	0.032 (0.030)	0.033 (0.029)
Black	−0.026 (0.028)	−0.013 (0.032)	0.007 (0.032)	0.004 (0.030)
Asian or Pacific Islander	−0.012 (0.039)	−0.068 (0.046)	−0.060 (0.044)	−0.054 (0.043)
Mixed race or other	−0.014 (0.033)	0.022 (0.038)	0.017 (0.037)	0.028 (0.036)
Hispanic or Latino ethnicity	0.005 (0.026)	−0.060** (0.030)	−0.054* (0.030)	−0.044 (0.029)
Income $50 000–$150 000	0.055** (0.021)	−0.066*** (0.025)	−0.058** (0.024)	−0.073*** (0.023)
Income >$150 000	0.068** (0.033)	−0.087** (0.038)	−0.079** (0.037)	−0.100*** (0.036)
Had no health insurance	−0.030 (0.037)	−0.003 (0.042)	−0.032 (0.041)	0.012 (0.040)
Married or cohabiting	−0.029 (0.020)	−0.004 (0.023)	−0.013 (0.022)	0.005 (0.022)
Household size	0.009 (0.008)	0.004 (0.009)	−0.001 (0.009)	0.007 (0.008)
Any children age 6–17 y	−0.038 (0.028)	0.030 (0.033)	0.030 (0.032)	0.038 (0.031)
Any children under 6 y	0.075** (0.032)	0.063* (0.037)	0.027 (0.036)	0.059* (0.035)
Nonelderly living with elderly	0.103*** (0.029)	0.055* (0.033)	0.056* (0.032)	0.048 (0.031)
Typically votes Republican	0.009 (0.021)	−0.015 (0.024)	−0.011 (0.023)	−0.018 (0.022)
Per-capita cases March–December 2020	−0.556 (0.449)	0.360 (0.519)	0.481 (0.505)	0.080 (0.488)
Number of pre-existing conditions	0.010 (0.009)	0.028*** (0.010)	0.025** (0.010)	0.028*** (0.010)
Was in fair or poor health	−0.012 (0.024)	0.047* (0.028)	0.041 (0.027)	0.058** (0.026)
Controls for baseline care consumption?	Yes	Yes	Yes	Yes
*P* value on the *F*-test for baseline care controls	<.001	.059	.005	.038
Observations	1480	1480	1480	1480
*R^2^*	0.08	0.06	0.05	0.08
Mean of dependent variable	0.142	0.199	0.184	0.174

Results from linear probability models; *P < .10, **P < .05, ***P < .01. The dependent variable in column 3 is an indicator for having a patient-initiated delay in care and reporting that COVID-19 concerns were an important reason for the delay. The dependent variable in column 4 is an indicator for having a patient-initiated delay in care and reporting that financial constraints, logistical constraints, or feeling overwhelmed was an important reason for the delay. All regressions include the following controls for baseline, pre-pandemic health care consumption (in a typical non-pandemic year): an indicator for whether the individual has at least 1 well visit per year, an indicator for having 1–2 sick visits per year, an indicator for 3+ sick visits per year, and an indicator for having no usual place for ambulatory care prior to the pandemic. Results from an analogous Logit specification are shown in Appendix Table A10.

Individuals from households including children under 6 years of age were 7.5 percentage points more likely to report experiencing an involuntary delay (*P* < .05), and respondents under age 65 years who live with an elderly household member were 10.3 percentage points more likely than those without elderly living companions to experience involuntary delays (*P* < .01).

A direct comparison of our main results with those in Callison and Ward^9^ is challenging because of differences in the 2 studies’ regression specifications and in the survey questions and reference periods defining involuntary delays. (The analysis in Callison and Ward^9^ is based on a question in the Current Population Survey [CPS] that is notably different from our survey's question about delays in care. Specifically, the CPS asks: “At any time in the last 4 weeks, did you or anyone in your household need medical care for something other than coronavirus, but not get it because of the coronavirus pandemic? Please include all adults and children in the household.” The fraction of their sample that experiences such involuntary delays is 0.028, which is much smaller than the fraction reporting involuntary delays in our sample, likely due to the difference in recall periods.) When we attempted to replicate the specification and dependent variable in that study as closely as our data will allow, we found many of the main results to be similar (see Appendix Table A7). However, the estimates from our preferred specification (Table 2, column 1), which includes controls for potential confounders like household income and baseline consumption of health care, indicate some important differences from their findings. For example, our results do not indicate a higher likelihood of involuntary delays for those in fair/poor health, those with health insurance, and older individuals. Table 2 reports the *P* values on the *F*-test for the joint significance of the controls for baseline care consumption; for involuntary delays, *P* < .001. Results for our preferred specification without the controls for baseline care utilization are displayed in Appendix Table A8.

### Determinants of patient-initiated delays in care

**Figure 1.**
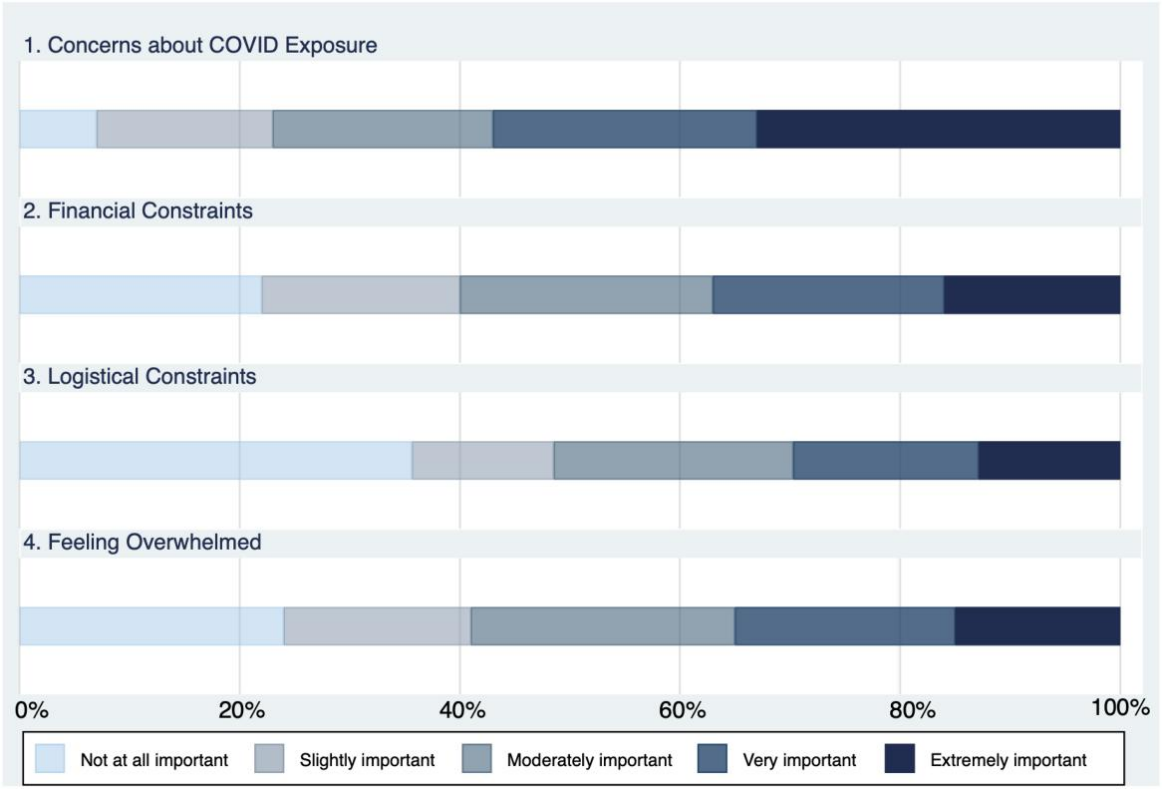
Importance of reasons for patient-initiated delays in medical care.

Over half (58%) of survey respondents who experienced pandemic-related delays in medical care reported initiating those delays themselves. Figure 1 displays how respondents rated the importance of these factors in causing them to delay or forgo care. It is striking that more than 90% of those who delayed or went without medical care reported that concerns about exposure to COVID-19 were a factor in their decision, and nearly 60% describe these concerns as “very important” or “extremely important.” Individuals’ ratings (on a scale from 1 to 5) of the importance of financial constraints, logistical constraints, and feeling overwhelmed were highly correlated (see Appendix Table A9). Therefore, we collapsed these 3 factors into 1 category and examined delays in care due to these reasons alongside delays due to concerns about COVID-19 exposure in the analysis that follows.

The second panel of Table 2 examines the association between individual characteristics and the following 3 outcomes: initiating delays in care for any reason (column 2), initiating a delay in care and reporting concerns about COVID-19 as an important reason (column 3), and initiating a delay in care and reporting financial difficulties, logistical constraints, and/or feelings of being overwhelmed as important factors (column 4).

Controlling for differences in baseline health care consumption, we document that several demographic characteristics are significantly related to the likelihood of delaying or going without care. For example, those who are older, are higher income, or are of Hispanic ethnicity had a lower probability of having initiated a delay in medical care during the pandemic (for all characteristics, *P* < .05). These results are similar for reductions in delaying medical care due to COVID-19 concerns and in delaying due to financial/logistical constraints or feeling overwhelmed.

On the other hand, respondents with young children (*P* < .10), nonelderly individuals who live with elderly household members (*P* < .10), and women (*P* < .05) were significantly more likely to have decided to delay medical care during the pandemic. For those living with young children, financial/logistical constraints and feelings of being overwhelmed by other life obligations were more important than concerns about COVID-19 exposure, which is consistent with our expectations, given that young children were perceived to be at little risk of serious health problems from COVID-19 during this period. Delays for those living with elderly household members, however, were driven by both concerns about COVID-19 and financial and logistical constraints. Women were significantly more likely than non-women to delay care due to concerns about COVID-19 exposure (*P* < .05), but not due to financial, logistical, or other constraints.

Not surprisingly, respondents’ decisions to delay care were associated with their health status. Those with more preexisting, chronic medical conditions were significantly more likely to have initiated delays in care (*P* < .01), as were those in fair or poor health (*P* < .10 for any patient-initiated delay; *P* < .05 for delaying due to financial/logistical constraints or feeling overwhelmed). These findings are consistent with evidence in Czeisler et al^1^ and Gertz et al,^15^ respectively, although those papers do not condition on baseline health care consumption. Last, it is worth noting that, after controlling for the included demographic characteristics, household income, and baseline consumption of health care, our measures of individuals’ political leanings and local COVID-19 conditions were not significantly related to their likelihood of having delayed or gone without medical care during the pandemic.

### Types of care affected by involuntary and patient-initiated delays

**Figure 2.**
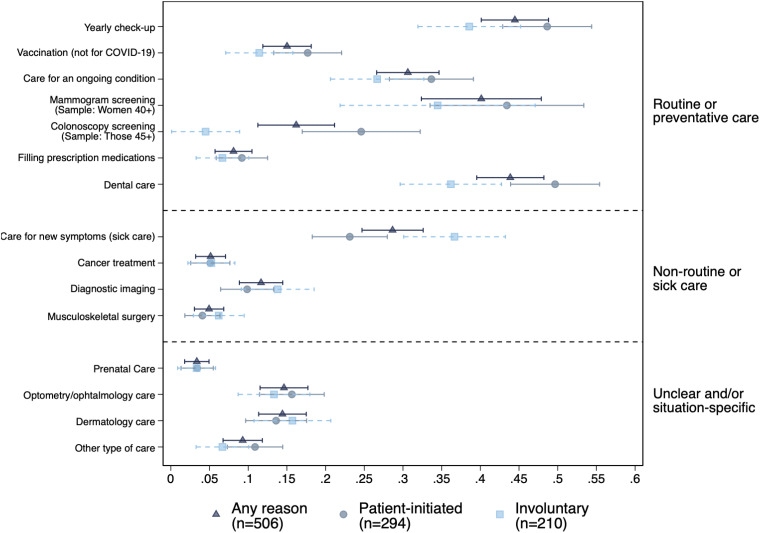
Types of care delayed by reason for delay.

Figure 2 presents evidence on which type(s) of medical care were most likely to have been delayed or missed entirely by respondents in our sample. Among those who experienced delays in care for any reason (indicated by the navy triangles and standard error bars), nearly one-half (44%) reported having delayed or gone without their annual checkup, and the same fraction reported interruptions in their dental care. Among those who were gender and age eligible, rates of delaying mammogram screening were also quite high, at 40%, and on average, those who delayed mammograms waited nearly 10.5 months before resuming in-person care. Over 30% reported having delayed care for an ongoing condition, and 28% delayed diagnosis and treatment for new symptoms. While rates of delay were lower for the remaining types of care in Figure 2, note that these types of care (eg, prenatal care) would be consumed by a smaller fraction of respondents even in non-pandemic times.

These averages mask important differences, however, between patients experiencing involuntary, “supply-side” delays (in dashed light blue) vs those who initiated their own “demand-side” delays in medical care (in solid medium blue). Those initiating their own delays in care appear to have been more likely to do so for forms of preventive care or routine care. For example, those initiating their own care delays were significantly more likely to delay or forgo dental care and colonoscopy screenings. While other differences are not statistically significant, the same pattern is upheld across all forms of preventive or routine care, including, for example, yearly checkups, vaccinations, and mammograms. On the other hand, those affected by involuntary care disruptions had significantly higher rates of delay for treatment of new symptoms than those who initiated their own care delays. We did not find significant differences in delays for other forms of nonroutine care or types of care that we were unable to classify as routine vs nonroutine.

### Consequences of delays in care

While the long-run health consequences of delayed and forgone medical care during the COVID-19 pandemic may not yet be known, our unique survey data can shed light on how those who experienced pandemic-related delays in care (either involuntary or patient-initiated) were impacted by these delays. The first 4 columns of Table 3 examine the impacts of experiencing a pandemic-related delay in medical care on self-reported health and on telemedicine utilization. The dependent variable in columns 1 and 2 is an indicator for the respondent reporting that their overall health worsened relative to before the pandemic, a question that was asked prior to any questions about pandemic-related delays in medical care. In columns 3 and 4, the outcome of interest is an indicator for their utilization of telemedicine having increased in 2020 and 2021, relative to 2019. All regressions include the same set of controls as in Table 2.

**Table 3.** Duration and consequences of delays in care.

	(1) Health worsened	(2) Health worsened	(3) Telemedicine increased	(4) Telemedicine increased	(5) Duration (months)	(6) Negative impact	(7) Regrets delay	(8) Delay again
Delayed care (any reason)	0.077*** (0.020)		0.085*** (0.029)					
Delayed care (availability)		0.081*** (0.024)		0.079** (0.034)	1.079 (0.783)	0.189*** (0.060)	0.098* (0.059)	0.011 (0.060)
Delayed care (COVID-19 risk)		−0.163*** (0.047)		0.025 (0.068)	2.091** (0.937)	−0.129* (0.071)	−0.046 (0.070)	0.096 (0.071)
Delayed care (financial, logistical, overwhelmed)		0.190*** (0.050)		−0.053 (0.072)	−0.858 (0.922)	0.201*** (0.071)	0.204*** (0.069)	0.067 (0.071)
Observations	1480	1480	1480	1480	425	506	506	506
*R* ^2^	0.11	0.12	0.04	0.04	0.13	0.21	0.12	0.17
Mean of dependent variable	0.155	0.155	0.496	0.496	8.845	0.449	0.308	0.619

Results from linear probability models; *P < .10, **P < .05, ***P < .01. All regressions include the same controls as in Table 2. Independent variable in columns 1 and 3 is an indicator for having delayed care for any reason. In columns 2 and 4–8, the 3 key independent variables are indicators for delaying care and indicating that availability was a reason, delaying care and indicating that COVID-19 risk was a reason, and delaying care and indicating that financial and logistical or feeling overwhelmed by other life obligations was a reason.

Overall, those who experienced any delays in medical care were nearly 8 percentage points (*P* < .001) more likely to report that their health worsened relative to before the pandemic (column 1). But again, this masks important differences between respondents who gave different reasons for their delays. Individuals who experienced involuntary delays in care or delays due to financial/logistical/overwhelmed constraints were significantly more likely to report that their health was worse relative to before the pandemic (*P* < .01), while those who delayed medical care due to concerns about contracting COVID-19 were less likely to believe their health had deteriorated (column 2, *P* < .01). Columns 3 and 4 reveal a positive association between delays in care and the use of telemedicine, although this is primarily driven by those whose care disruptions were caused by availability constraints.

Among those who experienced delays in care, how long did these delays last, and what were the consequences? Column 5 reveals that those who delayed care due to COVID-19 concerns had delays that lasted approximately 2 months longer, on average, than those for whom the risk of COVID-19 exposure was not a factor (*P* < .05). At the same time, these respondents were less likely to believe the delays had negatively impacted their health (column 6, *P* < .10). Respondents who experienced involuntary delays or delays due to financial/logistical/overwhelmed constraints were more likely to feel that their delays had negatively impacted their health (*P* < .01) and were more likely to “wish [they] had not delayed or gone without care as much as [they] did” (*P* < .01). Interestingly, those who delayed care due to risk of COVID-19 exposure were not significantly more likely to indicate that they would delay care again in light of the increase in COVID-19 cases during the Delta wave, perhaps because of the availability of vaccines. The relevant coefficient estimate is positive, however, as expected.

## Discussion

The COVID-19 pandemic caused many US adults to delay or go without needed medical care, often for long periods of time. More than one-third of our sample reported having delayed or gone without care during the pandemic, and the average delay in care lasted nearly 9 months. Such delays may have important implications for individuals’ long-run health. Indeed, nearly half (45%) of survey respondents who experienced a pandemic-related delay in medical care reported that the delay had negatively impacted their health.

This study helps shed light on some puzzling findings from prior research, like a positive relationship between having health insurance and experiencing involuntary delays in care^9^ or initiating delays in care due to COVID-19 concerns.^1^ Once we controlled for differences in respondents’ pre-pandemic propensity to consume medical care, we found no significant association between health insurance coverage and the likelihood of delaying care. Similarly, we consistently found that older adults were, if anything, less likely to experience delays in medical care, contributing to the mixed evidence on this question.^1,9,15^

Women's greater likelihood of delaying medical care due to COVID-19 concerns has also been documented by prior research^1^ but has gone mostly unexplained. Gender differences in preferences for risk-taking^16-18^ may help explain this result, particularly if the risk of COVID-19 infection felt more salient and immediate than the risk of delaying health care. Our survey asked respondents to complete a 30-item Domain-Specific Risk-Taking (DOSPERT) scale, which assesses risk-taking behavior in 5 different domains: recreational, financial, ethical, social, and health/safety.^17^ The average health/safety score for women in our sample was significantly lower (reflecting less willingness to take risk) than that for non-women (*P* < .001) as was the composite risk-taking score that combines all 5 domains (*P* < .001).

Perhaps surprisingly, we found that care-taking responsibilities played a role not only in causing patients to initiate delays in medical care but also in involuntary delays in care, with those living with young children or elderly adults being more likely to experience such delays. One explanation for this result may be that individuals reported that limited availability of care was the cause of their delay because they had trouble accessing care at times that did not conflict with their responsibilities at home. Our survey did not distinguish between those for whom needed medical care was completely unavailable and those who could not access care at a convenient time.

Finally, our results offer several key insights. First, policymakers and providers may consider targeting groups to ensure the resumption of care and help them “catch up” on missed care like well visits, routine cancer or cholesterol screenings, and care for ongoing conditions. Women and those with care-taking responsibilities (living with young children or elderly household members), younger individuals, patients with a greater number of preexisting conditions, and those in low-income households were more likely than others to choose to delay their own medical care and may be appropriate targets for such interventions.

Second, in the context of a similar public health event in the future, providers and policymakers should aim for more effective outreach to help patients evaluate the potentially significant risk of delaying preventive care, relative to the risk of potential exposure to an infectious disease. Patients in our sample who initiated their own delays in care were disproportionately likely to do so for types of care that are preventive or routine in nature, like dental care and colonoscopy screenings. This finding is worrisome, particularly in light of new evidence that many cancer diagnoses were missed during the COVID-19 pandemic.^3^

Third, our results suggest that providers should triage “sick care,” or care for new symptoms, when faced with capacity constraints due to a public health emergency or for other reasons, as was recommended by the Centers for Medicare and Medicaid Services in April of 2020. Survey respondents who experienced involuntary delays in care were particularly likely to experience delays in care for new symptoms and to report their health had worsened (8 percentage points, *P* < .01), that the delay had negatively impacted their health (19 percentage points, *P* < .01), and that they regretted the delay (10 percentage points, *P* < .10).

## Conclusion

Pandemic-related delays in medical care may have disparate and lasting impacts on the health of individuals. This study takes a comprehensive view of the determinants of involuntary and patient-initiated delays in care, providing new evidence on which groups may be most impacted. Controlling for differences in the propensity to consume health care across sociodemographic groups helps resolve some open questions from prior research. We found that patients who chose to delay medical care disproportionately did so for high-value preventive care. Our results point to negative impacts of care delays on self-reported health, but more research is needed to understand the longer-term impacts on patient outcomes.

## Supplementary Material

qxad057_Supplementary_Data
