# Supplementary material for: Involuntary and patient-initiated delays in medical care during the COVID-19 pandemic
Source: Health Aff Sch. 2023 Nov 2;1(5):qxad057. doi: 10.1093/haschl/qxad057 (PMC10986264; doi:10.1093/haschl/qxad057)
Supplement: qxad057_Supplementary_Data [file qxad057_Supplementary_Data.zip › Appendix_9.21.23.pdf]

Appendix

Table A1: Chronic Health Conditions

Survey question:
During the period from March 2020 through December 2020, did any of these health conditions apply to you? Please select all that apply.
Possible responses:
Chronic kidney disease
Chronic lung disease (e.g., COPD, asthma (moderate to severe), interstitial lung disease, cystic fibrosis, pulmonary hypertension)
Dementia or other neurological conditions
Diabetes (type 1 or type 2)
Down syndrome
Heart condition (e.g., heart failure, coronary artery disease, cardiomyopathies or hypertension)
HIV/AIDS
Immunocompromised state (not due to HIV/AIDS)
Liver disease
Overweight/obesity
Pregnancy
Sickle cell disease or thalassemia
Smoking (current smoker)
Smoking (former smoker)
Solid organ or blood stem cell transplant
Stroke or cerebrovascular disease
Substance use disorder
Physical impairment (sight, hearing, mobility)
Cognitive impairment
Mental illness
Learning disability
Other serious chronic condition
None of these

Table A2: Sample Mean Characteristics, by Type of Delay

	(1) Any Delay	(2) Involuntary Only	(3) Patient-Initiated Only	(4) Both Types
Demographics				
Age 18-24	0.162	0.162	0.107	0.181
Age 25-34	0.192	0.181	0.179	0.210
Age 35-44	0.219	0.233	0.143*	0.238
Age 45-54	0.111	0.105	0.119	0.114
Age 55-64	0.170	0.171	0.250	0.133
Age 65 and older	0.146	0.148	0.202	0.124
Woman	0.561	0.490	0.619**	0.610
White	0.652	0.643	0.714	0.638
Black	0.162	0.167	0.190	0.148
Asian or Pacific Islander	0.055	0.071	0.024	0.052
Mixed race or other	0.130	0.119	0.071	0.162
Hispanic or Latino ethnicity	0.198	0.219	0.143	0.200
Education				
High school degree or less	0.253	0.219	0.321*	0.262
Some college (<4-year)	0.478	0.490	0.440	0.481
Bachelor's degree or higher	0.269	0.290	0.238	0.257
Income				
Income < 50,000	0.350	0.233	0.476***	0.419
Income 50,000-150,000	0.534	0.619	0.476**	0.467
Income above 150,000	0.117	0.148	0.048**	0.114
Had no health insurance (Mar-Dec 2020)	0.057	0.033	0.060	0.081
Household Characteristics				
Married or cohabiting	0.534	0.557	0.488	0.529
Household size	3.093	3.219	2.488***	3.214
Household includes young children (< 6)	0.249	0.295	0.143***	0.248
Household includes children (6-17)	0.170	0.148	0.155	0.200
Household includes adults >65	0.267	0.338	0.155***	0.238
Non-elderly living with elderly	0.198	0.233	0.107**	0.195
Health Status				
Number of preexisting health conditions	1.743	1.705	1.548	1.871
Fair or poor health (prior to pandemic)	0.213	0.171	0.190	0.267
Baseline Healthcare Utilization				
Typically has at least one wellness visit per year	0.832	0.857	0.810	0.814
Sick visits never or less than once per year	0.316	0.271	0.440***	0.310
Sick visits 1-2 times per year	0.464	0.476	0.405	0.476
Sick visits 3 or more times per year	0.219	0.252	0.155*	0.214
Had no usual place for care (pre-pandemic)	0.202	0.171	0.167	0.248
Observations	506	210	84	210

Asterisks in column 3 indicate statistically significant difference between the value in column 2 and the value in column 3; * p<0.10, ** p<0.05, *** p<0.01. The samples in columns 2, 3, and 4 sum to 504 observations. This differs from the number of observations in column 1 because two individuals reported having delayed or forgone care but did not indicate that *any* of the factors we presented were important reasons for their delay. The number of observations in column 2 equals that in column 4 by coincidence only.

Table A3: Determinants of Pandemic-Related Delays in Medical Care
(Robustness check: Drop control for per-capita COVID cases in respondent's county)

	Involuntary Delays	*Patient-initiated Delays*		
	(1) Availability was Only Reason	(2) Any Patient-Initiated Delay	(3) Concerns re: Contracting COVID-19	(4) Financial, Logistical, Overwhelmed
Age 25-44	-0.036	-0.058	-0.065*	-0.056
	(0.032)	(0.037)	(0.036)	(0.035)
Age 45-64	-0.020	-0.075*	-0.085**	-0.110***
	(0.034)	(0.039)	(0.038)	(0.037)
Age 65 or older	-0.044	-0.110**	-0.113***	-0.142***
	(0.037)	(0.043)	(0.042)	(0.040)
Woman	-0.016	0.044**	0.044**	0.021
	(0.019)	(0.022)	(0.021)	(0.020)
Some college (<4-year)	0.030	-0.017	-0.005	0.002
	(0.022)	(0.025)	(0.024)	(0.023)
Bachelor's degree or higher	0.056**	0.015	0.031	0.032
	(0.026)	(0.031)	(0.030)	(0.029)
Black	-0.026	-0.013	0.007	0.004
	(0.028)	(0.032)	(0.032)	(0.030)
Asian or Pacific Islander	-0.009	-0.070	-0.062	-0.055
	(0.039)	(0.045)	(0.044)	(0.043)
Mixed race or other	-0.014	0.021	0.017	0.028
	(0.033)	(0.038)	(0.037)	(0.036)
Hispanic or Latino ethnicity	0.003	-0.059*	-0.053*	-0.044
	(0.026)	(0.030)	(0.030)	(0.029)
Income 50,000-150,000	0.056***	-0.067***	-0.059**	-0.073***
	(0.021)	(0.025)	(0.024)	(0.023)
Income above 150,000	0.066**	-0.086**	-0.078**	-0.100***
	(0.033)	(0.038)	(0.037)	(0.036)
Had no health insurance (Mar-Dec 2020)	-0.032	-0.002	-0.030	0.013
	(0.036)	(0.042)	(0.041)	(0.040)
Married or cohabiting	-0.029	-0.004	-0.012	0.006
	(0.020)	(0.023)	(0.022)	(0.022)
Household size	0.009	0.004	-0.001	0.007
	(0.008)	(0.009)	(0.009)	(0.008)
Any children ages 6-17 in household	-0.038	0.030	0.031	0.038
	(0.028)	(0.033)	(0.032)	(0.031)
Any children under 6 in household	0.073**	0.065*	0.029	0.059*
	(0.032)	(0.037)	(0.036)	(0.035)
Non-elderly living with elderly	0.102***	0.056*	0.057*	0.048
	(0.029)	(0.033)	(0.032)	(0.031)
Typically votes Republican	0.008	-0.014	-0.010	-0.018
	(0.021)	(0.024)	(0.023)	(0.022)
Number of preexisting conditions	0.010	0.028***	0.025**	0.028***
	(0.009)	(0.010)	(0.010)	(0.010)
Was in fair or poor health	-0.013	0.047*	0.042	0.058**
	(0.024)	(0.028)	(0.027)	(0.026)
Controls for baseline care consumption?	Yes	Yes	Yes	Yes
p-value on the F-test for baseline care controls	< 0.001	0.059	0.005	0.038
Observations	1480	1480	1480	1480
R^2	0.08	0.06	0.05	0.08
Mean of dependent variable	0.142	0.199	0.184	0.174

Results from linear probability models; * p<0.10, ** p<0.05, *** p<0.01. The dependent variable in column 3 is an indicator for having a patient-initiated delay in care and reporting that COVID concerns were an important reason for the delay. The dependent variable in column 4 is an indicator for having a patient-initiated delay in care and reporting that financial constraints, logistical constraints, or feeling overwhelmed was an important reason for the delay. All regressions include the following controls for baseline, pre-pandemic health care consumption (in a typical non-pandemic year): an indicator for whether the individual has at least one well visit per year, an indicator for having 1-2 sick visits per year, and indicator for 3+ sick visits per year, and an indicator for having no usual place for ambulatory care prior to the pandemic.

Table A4: Determinants of Pandemic-Related Delays in Medical Care
(Robustness check: Add state fixed effects)

	Involuntary Delays	*Patient-initiated Delays*		
	(1)	(2)	(3)	(4)
	Availability was Only Reason	Any Patient-Initiated Delay	Concerns re: Contracting COVID-19	Financial, Logistical, Overwhelmed
Age 25-44	-0.047	-0.058	-0.067*	-0.056
	(0.032)	(0.038)	(0.037)	(0.035)
Age 45-64	-0.027	-0.080**	-0.091**	-0.114***
	(0.035)	(0.040)	(0.039)	(0.038)
Age 65 or older	-0.057	-0.127***	-0.128***	-0.157***
	(0.038)	(0.044)	(0.043)	(0.041)
Woman	-0.011	0.043*	0.041*	0.025
	(0.019)	(0.022)	(0.022)	(0.021)
Some college (<4-year)	0.029	-0.008	0.005	0.012
	(0.022)	(0.026)	(0.025)	(0.024)
Bachelor's degree or higher	0.054**	0.029	0.044	0.048
	(0.027)	(0.031)	(0.030)	(0.029)
Black	-0.009	-0.008	0.009	0.010
	(0.030)	(0.035)	(0.034)	(0.033)
Asian or Pacific Islander	-0.015	-0.071	-0.059	-0.064
	(0.040)	(0.047)	(0.045)	(0.044)
Mixed race or other	-0.022	0.016	0.011	0.023
	(0.034)	(0.039)	(0.038)	(0.037)
Hispanic or Latino ethnicity	0.006	-0.060*	-0.054*	-0.042
	(0.027)	(0.031)	(0.030)	(0.029)
Income 50,000-150,000	0.047**	-0.066**	-0.059**	-0.073***
	(0.022)	(0.026)	(0.025)	(0.024)
Income above 150,000	0.044	-0.091**	-0.082**	-0.107***
	(0.034)	(0.040)	(0.039)	(0.037)
Had no health insurance (Mar-Dec 2020)	-0.012	-0.004	-0.032	0.015
	(0.037)	(0.043)	(0.042)	(0.041)
Married or cohabiting	-0.030	-0.008	-0.016	0.001
	(0.020)	(0.024)	(0.023)	(0.022)
Household size	0.006	0.001	-0.004	0.005
	(0.008)	(0.009)	(0.009)	(0.009)
Any children ages 6-17 in household	-0.022	0.023	0.023	0.028
	(0.029)	(0.033)	(0.032)	(0.031)
Any children under 6 in household	0.085***	0.062*	0.026	0.052
	(0.032)	(0.038)	(0.037)	(0.035)
Non-elderly living with elderly	0.108***	0.051	0.051	0.040
	(0.029)	(0.034)	(0.033)	(0.032)
Typically votes Republican	0.015	-0.002	0.002	-0.007
	(0.021)	(0.024)	(0.024)	(0.023)
Per-capita cases Mar-Dec 2020	-1.040	0.472	0.583	0.703
	(0.655)	(0.758)	(0.738)	(0.714)
Number of preexisting conditions	0.012	0.026**	0.023**	0.027***
	(0.009)	(0.010)	(0.010)	(0.010)
Was in fair or poor health	-0.008	0.055*	0.048*	0.063**
	(0.025)	(0.029)	(0.028)	(0.027)
Observations	1480	1480	1480	1480
R^2	0.11	0.09	0.09	0.10
Mean of dependent variable	0.142	0.199	0.184	0.174

Results from linear probability models; * p<0.10, ** p<0.05, *** p<0.01. The dependent variable in column 3 is an indicator for having a patient-initiated delay in care and reporting that COVID concerns were an important reason for the delay. The dependent variable in column 4 is an indicator for having a patient-initiated delay in care and reporting that financial constraints, logistical constraints, or feeling overwhelmed was an important reason for the delay. All regressions include the following controls for baseline, pre-pandemic health care consumption (in a typical non-pandemic year): an indicator for whether the individual has at least one well visit per year, an indicator for having 1-2 sick visits per year, and indicator for 3+ sick visits per year, and an indicator for having no usual place for ambulatory care prior to the pandemic.

Table A5: Determinants of Pandemic-Related Delays in Medical Care
(Robustness check: Drop respondents who spent < 10th percentile of time on survey)

	Involuntary Delays	*Patient-initiated Delays*		
	(1)	(2)	(3)	(4)
	Availability was Only Reason	Any Patient-Initiated Delay	Concerns re: Contracting COVID-19	Financial, Logistical, Overwhelmed
Age 25-44	-0.016	-0.112***	-0.117***	-0.109***
	(0.037)	(0.042)	(0.041)	(0.040)
Age 45-64	0.011	-0.131***	-0.136***	-0.166***
	(0.038)	(0.044)	(0.043)	(0.041)
Age 65 or older	-0.019	-0.183***	-0.177***	-0.213***
	(0.042)	(0.048)	(0.047)	(0.045)
Woman	-0.009	0.051**	0.045*	0.031
	(0.020)	(0.024)	(0.023)	(0.022)
Some college (<4-year)	0.025	-0.002	0.007	0.020
	(0.023)	(0.027)	(0.026)	(0.025)
Bachelor's degree or higher	0.049*	0.029	0.040	0.052*
	(0.028)	(0.033)	(0.032)	(0.031)
Black	0.003	-0.035	-0.019	-0.014
	(0.033)	(0.038)	(0.037)	(0.035)
Asian or Pacific Islander	-0.010	-0.094*	-0.072	-0.086*
	(0.044)	(0.051)	(0.050)	(0.047)
Mixed race or other	-0.041	0.005	0.001	0.013
	(0.036)	(0.042)	(0.041)	(0.039)
Hispanic or Latino ethnicity	0.029	-0.057*	-0.052	-0.038
	(0.029)	(0.034)	(0.033)	(0.032)
Income 50,000-150,000	0.035	-0.066**	-0.059**	-0.076***
	(0.023)	(0.027)	(0.026)	(0.025)
Income above 150,000	0.045	-0.117***	-0.098**	-0.134***
	(0.037)	(0.042)	(0.041)	(0.040)
Had no health insurance (Mar-Dec 2020)	-0.012	0.015	-0.021	0.036
	(0.039)	(0.046)	(0.045)	(0.043)
Married or cohabiting	-0.034	0.002	-0.006	0.013
	(0.021)	(0.025)	(0.024)	(0.023)
Household size	0.013	0.003	-0.003	0.008
	(0.009)	(0.010)	(0.010)	(0.009)
Any children ages 6-17 in household	-0.034	0.022	0.022	0.028
	(0.030)	(0.035)	(0.034)	(0.033)
Any children under 6 in household	0.070**	0.034	0.009	0.022
	(0.035)	(0.040)	(0.039)	(0.038)
Non-elderly living with elderly	0.117***	0.041	0.046	0.027
	(0.031)	(0.036)	(0.035)	(0.034)
Typically votes Republican	0.002	-0.003	-0.005	-0.008
	(0.022)	(0.025)	(0.025)	(0.024)
Per-capita cases Mar-Dec 2020	-0.884	0.153	0.487	0.409
	(0.686)	(0.795)	(0.777)	(0.743)
Number of preexisting conditions	0.011	0.029***	0.027**	0.031***
	(0.009)	(0.011)	(0.011)	(0.010)
Was in fair or poor health	0.010	0.025	0.020	0.036
	(0.026)	(0.031)	(0.030)	(0.029)
Observations	1332	1332	1332	1332
R^2	0.11	0.10	0.09	0.11
Mean of dependent variable	0.137	0.194	0.182	0.167

Results from linear probability models; * p<0.10, ** p<0.05, *** p<0.01. The dependent variable in column 3 is an indicator for having a patient-initiated delay in care and reporting that COVID concerns were an important reason for the delay. The dependent variable in column 4 is an indicator for having a patient-initiated delay in care and reporting that financial constraints, logistical constraints, or feeling overwhelmed was an important reason for the delay. All regressions include the following controls for baseline, pre-pandemic health care consumption (in a typical non-pandemic year): an indicator for whether the individual has at least one well visit per year, an indicator for having 1-2 sick visits per year, and indicator for 3+ sick visits per year, and an indicator for having no usual place for ambulatory care prior to the pandemic.

Table A6: Determinants of Pandemic-Related Delays in Medical Care
(Robustness check: Drop respondents who failed attention check late in survey)

	Involuntary Delays	*Patient-initiated Delays*		
	(1)	(2)	(3)	(4)
	Availability was Only Reason	Any Patient-Initiated Delay	Concerns re: Contracting COVID-19	Financial, Logistical, Overwhelmed
Age 25-44	-0.024	-0.090**	-0.106**	-0.087**
	(0.039)	(0.045)	(0.044)	(0.042)
Age 45-64	0.008	-0.131***	-0.144***	-0.165***
	(0.040)	(0.046)	(0.045)	(0.043)
Age 65 or older	-0.011	-0.160***	-0.159***	-0.189***
	(0.044)	(0.050)	(0.049)	(0.047)
Woman	-0.014	0.054**	0.053**	0.030
	(0.021)	(0.024)	(0.024)	(0.022)
Some college (<4-year)	0.020	-0.009	0.007	0.014
	(0.024)	(0.028)	(0.027)	(0.026)
Bachelor's degree or higher	0.034	0.026	0.045	0.054*
	(0.030)	(0.034)	(0.033)	(0.031)
Black	-0.039	-0.006	0.012	0.014
	(0.035)	(0.040)	(0.039)	(0.037)
Asian or Pacific Islander	-0.012	-0.106**	-0.088*	-0.097**
	(0.045)	(0.051)	(0.050)	(0.047)
Mixed race or other	-0.063	0.020	0.016	0.031
	(0.038)	(0.044)	(0.043)	(0.041)
Hispanic or Latino ethnicity	0.063**	-0.046	-0.032	-0.024
	(0.031)	(0.036)	(0.035)	(0.033)
Income 50,000-150,000	0.043*	-0.086***	-0.083***	-0.098***
	(0.024)	(0.028)	(0.027)	(0.026)
Income above 150,000	0.062	-0.113**	-0.093**	-0.133***
	(0.038)	(0.044)	(0.043)	(0.041)
Had no health insurance (Mar-Dec 2020)	0.012	-0.008	-0.046	0.019
	(0.041)	(0.047)	(0.046)	(0.043)
Married or cohabiting	-0.041*	0.025	0.017	0.035
	(0.022)	(0.026)	(0.025)	(0.024)
Household size	0.011	0.005	-0.002	0.011
	(0.010)	(0.011)	(0.011)	(0.010)
Any children ages 6-17 in household	-0.017	0.024	0.025	0.026
	(0.033)	(0.038)	(0.037)	(0.035)
Any children under 6 in household	0.109***	0.010	-0.014	-0.005
	(0.038)	(0.044)	(0.043)	(0.040)
Non-elderly living with elderly	0.134***	0.063*	0.079**	0.046
	(0.033)	(0.038)	(0.037)	(0.035)
Typically votes Republican	0.019	-0.014	-0.009	-0.019
	(0.023)	(0.026)	(0.025)	(0.024)
Per-capita cases Mar-Dec 2020	-1.077	0.146	0.399	0.456
	(0.702)	(0.806)	(0.785)	(0.746)
Number of preexisting conditions	0.009	0.025**	0.024**	0.025**
	(0.009)	(0.011)	(0.011)	(0.010)
Was in fair or poor health	0.029	0.056*	0.048	0.065**
	(0.028)	(0.032)	(0.031)	(0.030)
Observations	1209	1209	1209	1209
R	0.14	0.12	0.11	0.13
Mean of dependent variable	0.136	0.187	0.174	0.157

Results from linear probability models; * p<0.10, ** p<0.05, *** p<0.01. The dependent variable in column 3 is an indicator for having a patient-initiated delay in care and reporting that COVID concerns were an important reason for the delay. The dependent variable in column 4 is an indicator for having a patient-initiated delay in care and reporting that financial constraints, logistical constraints, or feeling overwhelmed was an important reason for the delay. All regressions include the following controls for baseline, pre-pandemic health care consumption (in a typical non-pandemic year): an indicator for whether the individual has at least one well visit per year, an indicator for having 1-2 sick visits per year, and indicator for 3+ sick visits per year, and an indicator for having no usual place for ambulatory care prior to the pandemic.

Table A7: Determinants of Involuntary Delays in Care
Specification and Dependent Variable Similar to Callison and Ward (2021)

	(1) Delayed due to Availability	(2) Callison and Ward (2021)
Age 18-24	0.061	0.0061
	(0.045)	(0.0049)
Age 35-44	0.001	0.0053
	(0.040)	(0.0045)
Age 45-54	-0.095**	0.0165***
	(0.044)	(0.0048)
Age 55-64	-0.059	0.0254***
	(0.043)	(0.0048)
Age 65-74	-0.182***	0.0385***
	(0.043)	(0.0055)
Age 75 or older	-0.148**	0.0345***
	(0.060)	(0.0069)
Woman	0.013	0.0091***
	(0.024)	(0.0028)
White-Hispanic	0.004	-0.0108***
	(0.053)	(0.0042)
Black	-0.034	-0.0107**
	(0.035)	(0.0049)
Asian or Pacific Islander	-0.044	-0.0289***
	(0.051)	(0.0048)
Mixed race or other	0.010	-0.0178**
	(0.041)	(0.0072)
Less than high school degree	-0.038	-0.0109**
	(0.072)	(0.0045)
Bachelor's degree or higher	0.081***	0.0128***
	(0.029)	(0.0033)
Had no health insurance	-0.103**	-0.0101**
	(0.045)	(0.0047)
Jan 20 health status - very good	-0.046	0.0066*
	(0.040)	(0.00304)
Jan 20 health status - good	-0.011	0.0100***
	(0.038)	(0.0038)
Jan 20 health status - fair	0.027	0.0437***
	(0.045)	(0.0068)
Jan 20 health status - poor	0.117*	0.0534***
	(0.068)	(0.0124)
N	1,480	34,849
Mean of d.v. for reference category	0.345	0.028

Results from linear probability models; * p<0.10, ** p<0.05 *** p<0.01. The dependent variable in Callison and Ward (2021) is based on a question in the Current Population Survey (CPS) that is notably different from our survey's question about delays in care. Specifically, the CPS asks: "At any time in the last 4 weeks, did you or anyone in your household need medical care for something other than Coronavirus, but not get it because of the Coronavirus pandemic? Please include all adults and children in the household." The reference period is March–May 2020, whereas our dependent variable identifies delays in care during the period from March 2020–October 2021.

Table A8: Determinants of Pandemic-Related Delays in Medical Care
Dropping Baseline Care Controls

	Involuntary Delays	*Patient-initiated Delays*		
	(1) Availability was Only Reason	(2) Any Patient-Initiated Delay	(3) Concerns re: Contracting COVID-19	(4) Financial, Logistical, Overwhelmed
Age 25-44	-0.035	-0.057	-0.063*	-0.054
	(0.032)	(0.037)	(0.036)	(0.035)
Age 45-64	-0.024	-0.078**	-0.087**	-0.113***
	(0.034)	(0.039)	(0.038)	(0.037)
Age 65 or older	-0.055	-0.114***	-0.117***	-0.150***
	(0.037)	(0.043)	(0.042)	(0.040)
Woman	-0.011	0.047**	0.048**	0.025
	(0.019)	(0.022)	(0.021)	(0.020)
Some college (<4-year)	0.027	-0.015	-0.003	0.000
	(0.022)	(0.025)	(0.024)	(0.023)
Bachelor's degree or higher	0.064**	0.022	0.040	0.038
	(0.027)	(0.030)	(0.030)	(0.029)
Black	-0.022	-0.009	0.010	0.006
	(0.028)	(0.032)	(0.032)	(0.030)
Asian or Pacific Islander	-0.021	-0.073	-0.066	-0.060
	(0.040)	(0.046)	(0.044)	(0.043)
Mixed race or other	-0.019	0.017	0.010	0.026
	(0.033)	(0.038)	(0.037)	(0.035)
Hispanic or Latino ethnicity	0.021	-0.048	-0.040	-0.034
	(0.026)	(0.030)	(0.029)	(0.028)
Income 50,000-150,000	0.054**	-0.066***	-0.058**	-0.074***
	(0.022)	(0.025)	(0.024)	(0.023)
Income above 150,000	0.072**	-0.084**	-0.075**	-0.099***
	(0.033)	(0.038)	(0.037)	(0.036)
Had no health insurance (Mar-Dec 2020)	-0.058	-0.025	-0.061	-0.002
	(0.036)	(0.041)	(0.040)	(0.038)
Married or cohabiting	-0.027	-0.002	-0.010	0.006
	(0.020)	(0.023)	(0.022)	(0.022)
Household size	0.009	0.004	-0.001	0.007
	(0.008)	(0.009)	(0.009)	(0.008)
Any children ages 6-17 in household	-0.027	0.037	0.039	0.045
	(0.028)	(0.033)	(0.032)	(0.031)
Any children under 6 in household	0.097***	0.077**	0.043	0.074**
	(0.032)	(0.037)	(0.036)	(0.034)
Non-elderly living with elderly	0.109***	0.060*	0.062*	0.052*
	(0.029)	(0.033)	(0.032)	(0.031)
Typically votes Republican	0.007	-0.017	-0.013	-0.019
	(0.021)	(0.024)	(0.023)	(0.022)
Per-capita cases Mar-Dec 2020	-0.561	0.349	0.474	0.076
	(0.453)	(0.520)	(0.507)	(0.489)
Number of preexisting conditions	0.018**	0.033***	0.031***	0.032***
	(0.009)	(0.010)	(0.010)	(0.009)
Was in fair or poor health	-0.003	0.051*	0.047*	0.065**
	(0.024)	(0.028)	(0.027)	(0.026)
Controls for baseline care consumption?	No	No	No	No
Observations	1480	1480	1480	1480
R^2	0.06	0.05	0.05	0.07
Mean of dependent variable	0.142	0.199	0.184	0.174

Results from linear probability models; * p<0.10, ** p<0.05, *** p<0.01. The dependent variable in column 3 is an indicator for having a patient-initiated delay in care and reporting that COVID concerns were an important reason for the delay. The dependent variable in column 4 is an indicator for having a patient-initiated delay in care and reporting that financial constraints, logistical constraints, or feeling overwhelmed was an important reason for the delay.

Table A9: Correlations between Ratings of the Importance of Reasons for Initiating Delays in Care

	Concerns about contracting COVID-19	Financial constraints	Logistical constraints	Feeling overwhelmed
Concerns about contracting COVID-19	1.0000			
Financial constraints	0.0333	1.0000		
Logistical constraints	0.0906	0.5313	1.0000	
Feeling overwhelmed	0.0557	0.5797	0.5892	1.0000

Table A10: Determinants of Pandemic-Related Delays in Medical Care
(Estimates from a Logit model)

	Involuntary Delays	*Patient-initiated Delays*		
	(1) Availability was Only Reason	(2) Any Patient-Initiated Delay	(3) Concerns re: Contracting COVID-19	(4) Financial, Logistical, Overwhelmed
Age 25-44	-0.323	-0.358	-0.402*	-0.367
	(0.266)	(0.228)	(0.232)	(0.231)
Age 45-64	-0.159	-0.475*	-0.555**	-0.762***
	(0.284)	(0.246)	(0.251)	(0.258)
Age 65 or older	-0.451	-0.772***	-0.800***	-1.194***
	(0.328)	(0.284)	(0.288)	(0.313)
Woman	-0.137	0.296**	0.316**	0.161
	(0.167)	(0.146)	(0.150)	(0.155)
Some college (<4-year)	0.294	-0.132	-0.051	-0.022
	(0.203)	(0.165)	(0.171)	(0.178)
Bachelor's degree or higher	0.464**	0.093	0.206	0.237
	(0.235)	(0.197)	(0.203)	(0.211)
Black	-0.217	-0.077	0.052	0.040
	(0.243)	(0.213)	(0.216)	(0.222)
Asian or Pacific Islander	-0.079	-0.467	-0.440	-0.397
	(0.327)	(0.332)	(0.345)	(0.347)
Mixed race or other	-0.128	0.141	0.118	0.194
	(0.290)	(0.238)	(0.246)	(0.246)
Hispanic or Latino ethnicity	0.058	-0.399**	-0.375*	-0.333
	(0.220)	(0.199)	(0.203)	(0.204)
Income 50,000-150,000	0.517**	-0.432***	-0.408**	-0.546***
	(0.201)	(0.159)	(0.164)	(0.170)
Income above 150,000	0.599**	-0.577**	-0.560**	-0.754***
	(0.284)	(0.257)	(0.265)	(0.274)
Had no health insurance (Mar-Dec 2020)	-0.469	0.003	-0.202	0.102
	(0.420)	(0.272)	(0.296)	(0.277)
Married or cohabiting	-0.231	-0.044	-0.105	0.005
	(0.180)	(0.151)	(0.155)	(0.161)
Household size	0.076	0.024	-0.009	0.043
	(0.063)	(0.056)	(0.059)	(0.058)
Any children ages 6-17 in household	-0.344	0.206	0.208	0.296
	(0.254)	(0.209)	(0.215)	(0.217)
Any children under 6 in household	0.439*	0.402*	0.198	0.411*
	(0.253)	(0.229)	(0.240)	(0.236)
Non-elderly living with elderly	0.659***	0.317	0.349*	0.299
	(0.212)	(0.199)	(0.205)	(0.208)
Typically votes Republican	0.094	-0.118	-0.101	-0.179
	(0.184)	(0.162)	(0.167)	(0.177)
Per-capita cases Mar-Dec 2020	-5.753	2.561	3.517	0.699
	(4.102)	(3.468)	(3.567)	(3.736)
Number of preexisting conditions	0.056	0.148**	0.135**	0.164***
	(0.067)	(0.058)	(0.060)	(0.060)
Was in fair or poor health	-0.054	0.301*	0.275	0.419**
	(0.216)	(0.172)	(0.177)	(0.179)
Controls for baseline care consumption?	Yes	Yes	Yes	Yes
Observations	1480	1480	1480	1480
Mean of dependent variable	0.142	0.199	0.184	0.174

Results from logit models; * p<0.10, ** p<0.05, *** p<0.01. The dependent variable in column 3 is an indicator for having a patient-initiated delay in care and reporting that COVID concerns were an important reason for the delay. The dependent variable in column 4 is an indicator for having a patient-initiated delay in care and reporting that financial constraints, logistical constraints, or feeling overwhelmed was an important reason for the delay. All regressions include the following controls for baseline, pre-pandemic health care consumption (in a typical non-pandemic year): an indicator for whether the individual has at least one well visit per year, an indicator for having 1-2 sick visits per year, and indicator for 3+ sick visits per year, and an indicator for having no usual place for ambulatory care prior to the pandemic.
